# Randomized clinical trials in orthodontics are rarely registered *a priori* and often published late or not at all

**DOI:** 10.1371/journal.pone.0182785

**Published:** 2017-08-04

**Authors:** Spyridon N. Papageorgiou, Georgios N. Antonoglou, George K. Sándor, Theodore Eliades

**Affiliations:** 1 Clinic of Orthodontics and Pediatric Dentistry, Center of Dental Medicine, University of Zurich, Zurich, Switzerland; 2 Institute of Dentistry, Department of Oral and Maxillofacial Surgery, University of Oulu, Oulu, Finland; 3 Department of Periodontology and Implant Biology, Dental School, Aristotle University of Thessaloniki, Thessaloniki, Greece; 4 BioMediTech, Institute of Bioscience and Technology, University of Tampere, Tampere, Finland; University of Washington, UNITED STATES

## Abstract

*A priori* registration of randomized clinical trials is crucial to the transparency and credibility of their findings. Aim of this study was to assess the frequency with which registered and completed randomized trials in orthodontics are published. We searched ClinicalTrials.gov and ISRCTN for registered randomized clinical trials in orthodontics that had been completed up to January 2017 and judged the publication status and date of registered trials using a systematic protocol. Statistical analysis included descriptive statistics, chi-square or Fisher exact tests, and Kaplan-Meier survival estimates. From the 266 orthodontic trials registered up to January 2017, 80 trials had been completed and included in the present study. Among these 80 included trials, the majority (76%) were registered retrospectively, while only 33 (41%) were published at the time. The median time from completion to publication was 20.1 months (interquartile range: 9.1 to 31.6 months), while survival analysis indicated that less than 10% of the trials were published after 5 years from their completion. Finally, 22 (28%) of completed trials remain unpublished even after 5 years from their completion. Publication rates of registered randomized trials in orthodontics remained low, even 5 years after their completion date.

## Introduction

Randomized clinical trials are the gold standard in comparative effectiveness research, due to their explicit methods, internal validity, and transparency. Crucial to this transparency is the provision of an *a priori* designed protocol that delineates each trial aspect. This can also be used to compare the original trial plan with its subsequent publication [1] to ascertain the trial’s original scope, as well as minimize the risk of data dredging. Furthermore, *a priori* registration of clinical trials can safeguard against dangers like delayed publication or non-publication of trials, selective reporting of outcomes, per protocol rather than intention-to-treat analyses, and trial overlaps among papers included within systematic reviews [1–4]. Such *a priori* registered trial protocols can be either published as standalone publications [5] and/or be registered in freely accessible online repositories [6].

One such freely accessible online repository is ClinicalTrials.gov (https://clinicaltrials.gov/), a trial registry and results database maintained by the US National Library of Medicine. Although there is no formal requirement that all funded trials must be registered in ClinicalTrials.gov, the International Committee of Medical Journal Editors (ICMJE) began from 2005 requiring trial registration as a prerequisite for publication in any of its member journals [7]. This led to a considerable increase of trials being registered, which now reach several hundreds of new trials being registered each week [8]. Another well-known registry for clinical trials in medicine is the International Standard Randomised Controlled Trial Number (ISRCTN) registry (http://www.isrctn.com/), which contains the basic set of data items deemed essential to describe a study at inception, as per the requirements set out by the World Health Organization, the International Clinical Trials Registry Platform, and the ICMJE guidelines. These two registries represent the two major recipients of trial registrations worldwide [8], while all trial records in these databases are freely accessible and have been assigned a unique trial identifier, thereby enabling linking trial registrations to their subsequent publications.

However, existing research shows that the results of studies are often not shared publicly in a timely way and that between 25% and 50% of clinical trials remain unpublished even several years after completion [9–11]. There are many possible reasons behind the delayed or non-publication of results from clinical trials, including lack of incentive to disseminate negative findings, time constraints, limited resources, changing research interests, or even failure to have an article accepted by a journal. Importantly, time-lags in the dissemination of results may have adverse consequences for the practice of evidence based medicine and for public health [12]. The non-publication of trial results also violates an ethical obligation that trial investigators have towards trial participants [13]. Even if trials do eventually get published years later, it may be too late, and the results may have less relevance because of the rapid changes in the landscape of evidence.

Although several studies have estimated the proportion of incomplete or selective reporting in various medical specialties [14–17], no such assessments exist in orthodontics. A previous study [18] provided an overview of registered trials in orthodontics up to 2014, but was limited to descriptive analysis of their registrations and did not assess which were eventually published. Aim of the present study was to assess the publication fate of registered orthodontic randomized clinical trials and to assess characteristics related to their publication timing.

## Materials and methods

### Study sample

The protocol for the present study was drafted prior to study initiation, but was not registered in PROSPERO, as it did not meet PROSPERO’s prerequisite of having clinical connection to the trials’ results. For this study two authors (SNP and GNA) searched independently two online registries for randomized trials, ClinicalTrials.gov and ISRCTN, up to January 2017. As we intended to assess the publication fate of these trials, only registrations of trials marked in the registries as completed were included. Registrations of non-randomized or non-orthodontic trials were excluded, while any registrations pertaining to the same trial were grouped together.

The same two authors (SNP and GNA) subsequently searched independently in January 2017 for any publications originating from the completed registered trials using a previous comprehensive locating protocol [19]. Initially, the publication field of the trial protocol registration was searched for any linked references. Secondly, the unique trial identifier assigned within each registration was used to search the web through Google (https://www.google.com), Google Scholar (https://scholar.google.com/), and MEDLINE via Pubmed (https://www.ncbi.nlm.nih.gov/pubmed/). Finally, if no corresponding publications could be traced, the names of all investigators listed in the trial protocol as principal investigator or otherwise were used for searches in MEDLINE, combining them with the keyword orthodon* (Table A in S1 File). Manually-identified publications were judged as originating from a specific trial registration, if either (i) the unique registration identifier was reported within the trial publication or (ii) the used author names overlapped between protocol and publication, the trial was randomized and on the same subject as the protocol, and patient enrollment in the publication matched the trial protocol description. Only publications from scientific journals were included, while trial publications in the form of theses/dissertations were excluded, as they follow considerably different publication pathways and are not subject to the same peer review procedures as journal papers. The trial’s Pubmed Unique Identifier (PMID) (or manual unique identifiers in case that didn’t exist) was assigned to each identified trial publication for identification purposes.

### Data extraction

The same two authors (SNP and GNA) independently extracted data on the level of trial registration and trial publication, using pre-defined and calibrated extraction forms. Extracted data from the trial’s registration included registry, registration number, clinal setting (university clinic, hospital, or private practice), geographic information (country and continent), trial start- and end-date, sponsor (internal, government, commercial, or other), registration’s timing (registered before or after trial initiation [20]), trial nature (single-center of multicenter), outcome type (patient-reported or investigator-assessed), age of eligible patients (children, adults, or mixed), and trial size (arbitrarily categorized as ≥ or < 100 patients per trial arm based on empirical data [21]). Extracted data from the trial’s publication included, PMID, publication date, number of identified publications from the same trial, journal name, journal publication type (with or without electronic publication ahead of print), and trial findings (judged from the abstract as positive, not positive, or unclear). Information about the affiliation and geographical location of a trial was based on the person listed as principal investigator of each trial or the responsible party, if no principal investigator was listed. We measured the time from trial completion to publication by calculating the duration of time (in months). The trial completion date was determined from the trial’s registration, while the earliest date a trial emerged in MEDLINE was adopted as its publication date. For two trials that were not listed in MEDLINE publication date was the first day of the journal publication issue’s month. If a trial publication pertained to preliminary results of a trial and not the final results of the trial’s primary outcome, the trial was coded as unpublished. For all trials without any identified publications we calculated the follow-up time as the duration in months between trial completion and end date of our study (January 12, 2017).

### Statistical analysis

We conducted a descriptive analysis calculating frequencies for binary outcomes or medians, Interquartile Ranges (IQRs), and ranges for continuous outcomes, due to skewness of the latter. Simple differences in the trial publication rate and completion-to-publication time according to the various descriptive characteristics were investigated with chi-square /Fisher exact and Kruskal-Wallis tests, respectively. For the analyses, geographic regions (continent) were merged together, if less than 10 trials to avoid data thinning. In addition, we evaluated publication fate of included registered trials using Kaplan-Meier analysis after checking for assumptions and assessed influencing factors with graphical inspection and log-rank tests. For the survival analyses the completion-to-publication time for published trials (or the completion-to-January 2017 time for unpublished trials) were used as follow-up periods, while all trials that remained unpublished were censored. All analyses of data (S1 Dataset) were performed in Stata version SE 14 (StataCorp LP, College Station, TX) with a level of significance set at 5%.

## Results

**Fig 1:**
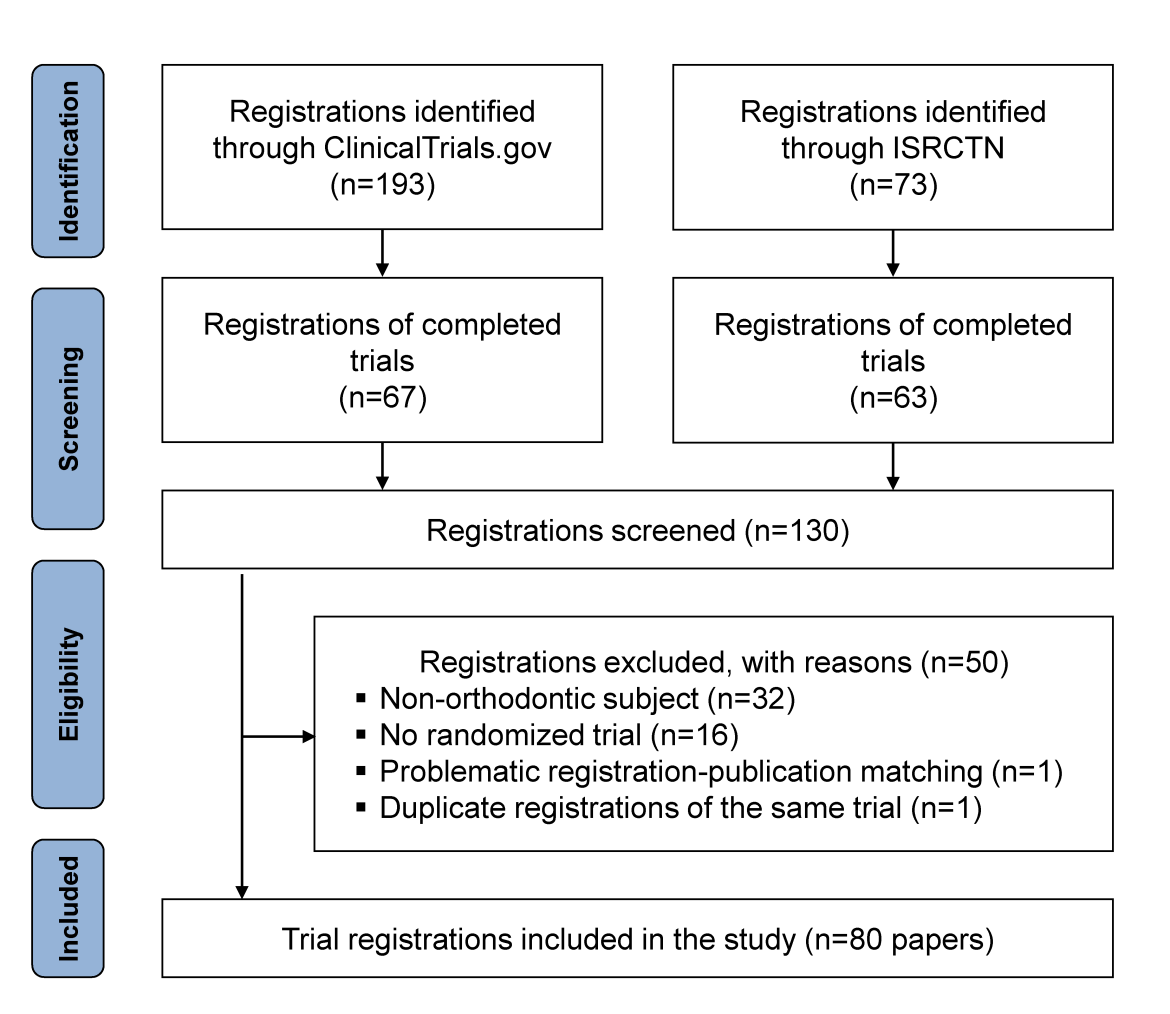
Flowdiagram of the study selection.

We identified a total of 266 registered trials in orthodontic, 130 of which were completed in January 2017, and were assessed for eligibility in the present study (Fig 1; Table B in S1 File). Finally, a total of 50 registered trials were judged as ineligible, leaving 80 registered trials that were finally included in this study. These pertained to 80 randomized trials, the majority (59%) of which originated from Europe and specifically from the United Kingdom (44%). They were initiated between 1997–2015 and were completed between 2003–2016 (Table 1).

**Table 1:** Demographics of the included registered trial protocols.

		N^a^	N	%
Register	ClinicalTrials.gov	80	45	56%
	ISRCTN		35	44%
Country	Austria	80	1	1%
	Belgium		1	1%
	Brazil		5	6%
	Canada		2	3%
	China		1	1%
	Colombia		1	1%
	Egypt		1	1%
	France		2	3%
	Germany		4	5%
	Hong Kong		1	1%
	India		3	4%
	Iran		1	1%
	Italy		2	3%
	Kuwait		1	1%
	Sweden		1	1%
	Syria		4	5%
	United Kingdom		35	44%
	Uinted States of America		12	15%
	Not reported		2	3%
Setting	University	80	63	79%
	Private		1	1%
	Hospital		16	20%
Continent	Europe	80	47	59%
	Asia		11	14%
	North America		14	18%
	South America & Africa		8	10%

ISRCTN = International Standard Randomised Controlled Trial Number; N^a^ = eligible trials trials included in the assessment of this factor; N = trials in each factor category.

From the identified trials, half of them (50%) had no external financial support, while only the minority (8%) included multiple trial sites (Table 2). The median number of planned patients to be enrolled in the trial was 50 patients (IQR: 30–90 patients; Table 3), while only 23% of the trials had a large sample size (≥ 100 patients). Interestingly, only 24% of trial protocols were registered prospectively and the vast majority of trials (76%) were registered after trial initiation.

**Table 2:** Characteristics of the included registered trial protocols (binary variables).

Factor	Category	N^a^	N	%
Sponsor	Internal	80	40	50%
	Government		21	26%
	Commercial		10	13%
	Other		9	11%
Registration	Prospective	80	19	24%
	Retrospective		61	76%
Outcome	Patient-reported	79	6	8%
	Investigator-asserted		73	92%
Large trial (≥100 patients per trial arm)	Yes	71	5	7%
	No		66	93%
Multicenter trial	Yes	80	6	8%
	No		74	93%
Eligible patients	Children	80	29	36%
	Adults		8	10%
	Mixed		29	36%
	Not specified		14	18%
Trial published	Yes	80	33	41%
	No		47	59%
Publication fate after trial completion	Within 12 months	33	10	30%
	Within 24 months		18	55%
	Within 36 months		27	82%
	Within 48 months		29	88%
	Within 60 months		30	91%
Publications from registered trial protocol	None	80	47	59%
	One		25	31%
	Two		5	6%
	Three		3	4%
Trials remaining unpublished after 5 years	Yes	80	22	28%
	No		58	72%
Journal	Am J Orthod Dentofacial Orthop	33	8	24%
	Angle Orthod		1	3%
	Br J Oral Maxillofac Surg		1	3%
	Caries Res		1	3%
	Eur J Orthod		6	18%
	Int J Paediatr Dent		1	3%
	J Am Dent Assoc		1	3%
	J Clin Diagn Res		1	3%
	J Contemp Dent Pract		1	3%
	J Dent Res		3	9%
	J Indian Orthod Soc		1	3%
	J Orthod		5	15%
	J Pharm Biomed Sci		1	3%
	Photomed Laser Surg		1	3%
	Sci Rep		1	3%
Journal type	Specialty (orthodontic)	33	21	64%
	Non-specialty		12	36%
Journal with E-publication ahead of print	Yes	33	18	55%
	No		15	45%
Trial results	Positive	33	15	42%
	Not positive		14	45%
	Unclear		4	12%

N^a^ = eligible trials trials included in the assessment of this factor; N = trials in each factor category.

**Table 3:** Cross-tabulation of subsequent trial publication and protocol characteristics.

Factor	Category	Published N (%)	Unpublished N (%)	P*
Registry	ClinicalTrials.gov	20 (44%)	25 (56%)	0.51
	ISRCTN	13 (37%)	22 (63%)	
Sponsor	Internal	14 (35%)	26 (65%)	0.30
	Government	10 (48%)	11 (52%)	
	Commercial	3 (30%)	7 (70%)	
	Other	6 (67%)	3 (33%)	
Affiliation	University	26 (41%)	37 (59%)	1.00
	Private practice	0 (0%)	1 (100%)	
	Hospital	7 (78%)	9 (22%)	
Continent	Europe	20 (43%)	27 (57%)	0.30
	Asia	8 (73%)	3 (27%)	
	N. America	2 (14%)	12 (86%)	
	S. America & Africa	3 (38%)	5 (62%)	
Registration	Prospectively	26 (43%)	35 (57%)	0.66
	Retrospectively	7 (37%)	12 (63%)	
Outcome	Patient-reported	31 (42%)	42 (58%)	1.00
	Doctor-assessed	2 (33%)	4 (67%)	
Multicenter trial	Yes	2 (33%)	4 (67%)	1.00
	No	31 (42%)	42 (58%)	
Patients	Children	14 (48%)	15 (52%)	0.07
	Adults	2 (25%)	6 (75%)	
	Mixed	15 (52%)	14 (48%)	
	Not specified	2 (14%)	12 (86%)	
Large trial	Yes	4 (80%)	1 (20%)	0.17
	No	28 (42%)	38 (58%)	

ISRCTN = International Standard Randomised Controlled Trial Number.

*from chi-square (or Fisher’s exact test, when more than 20% of the cells have expected frequency less than five or at least one is null)

As far as publication fate of the registered trials is concerned, only 33 (41%) of the 80 identified completed trials including at least 3839 patients had been published in journals at the time this study was completed, with 10% of trials being published in multiple papers (Table 2). The majority of registered trials were published in specialty journals and in journals that provided electronic publication ahead of print option (64% and 55% respectively). Finally, about equal parts of the 33 published trials reported positive and non-positive findings (42% and 45%, respectively), while results were inconclusive in the remaining 4 trials (12%). No association between publication rate and characteristics of the registered trials could be found (Table 3). The single exception was the geographic location of the trial, with registered trials from North America and Africa being published less often than trials from other continents. However, the remaining 47 (59%) registered and completed trials, including at least 2355 randomized patients, remained unpublished at the time of the present study.

Among the 33 completed trials that were eventually published, 10 (30%), 18 (55%), 27 (82%), 29 (88%), and 30 (91%) of them were published within 12, 24, 36, 48, and 60 months after trial completion, respectively (Table 2). The median time from completion to publication was 20.1 months with an IQR of 9.1–31.6 months) (Table 4). Considerable differences in the completion-to-publication time were found according to registry, sponsor type, patient age, and journal (Table C in S1 File), with trials from ClinicalTrials.gov, internally sponsored, with adult or mixed patient populations, and trials submitted in non-specialty journals were published faster than the rest.

**Table 4:** Characteristics of the included registered trial protocols (continuous variables).

Outcome	Extracted from	N^a^	Mean (SD)	Median (IQR)	Range
Reported sample size (patients)	Protocol	71	87.2 (138.4)	50.0 (30.0–90.0)	8.0–1000.0
Trial duration (months)	Protocol	80	29.5 (22.4)	24.3 (15.2–36.5)	0.9–124.8
Completion-to-publication (months)	Protocol / publication	33	24.5 (18.0)	20.1 (9.1–31.6)	1.8–65.0
Mean follow-up of trial protocol for the present study (months)	Protocol / publication	80	47.7 (45.8)	28.4 (13.0–64.7)	1.8–166.6

SD = standard deviation; IQR = interquartile range (Q1-Q3).

The Kaplan-Meier estimates of the publication fate for the 80 included trials indicated that most of them were published during the first 24 or 48 months after completion (Figure A in S2 File). On the other side, about 60 months (five years) after completion no considerable publication activity was seen. Differences in the survival curves can be seen in Figures B-M in S2 File. Trials registered in ClinicalTrials.gov seemed to be published faster and more often than trials registered in ISRCTN (Figure B in S2 File), while considerable differences in the publication fate were seen across continents (Figure E in S2 File). Additionally, trials with adult patients or trials with unspecified patient populations seemed to be published less or more slowly than trials on children (Figure I in S2 File). Finally, among the 33 published trials, trials in non-specialty journals seemed to get published faster than trial publications in specialty journals (Figure K in S2 File).

As the majority (30 out of 33 trials; 91%) of completed and published trials were published within 5 years from their completion (Table 2), a free-period of 5 years from completion was allowed for all 80 identified and completed trials. However, about one third (n = 22; 28%) of completed trials still remained unpublished, even though 5 years had elapsed from trial completion, with small chances of the trial finally getting published.

## Discussion

### Summary of evidence

The present study assessed the publication fate as well as the completion-to-publication time of all randomized trials in orthodontics that had been registered in ClinicalTrials.gov and ISRCTN and had been filed as ‘completed’ up to January 2017.

The results indicated that at the time of completion of the present study (January 2017) 59% of all registered and completed trials had not been published, which corresponded to at least 2355 included patients (some trials did not report the planned patient sample). This agrees with similar studies on the biomedical literature that report non-publication rates of 29% [15], 34% [16], 50% [17], and 54% [14].

As far as time to publication is concerned, the results indicated that the median completion-to-publication time was 20.1 months (1.6 years). This also is similar to the findings of previous investigations, which report a median completion-to-publication time of 28.8 months (2.4 years) [22]. Additionally, survival analysis indicated that most of the trials were published within the first 24–48 months (2–4 years) after the trial’s completion. After about 60 months (5 years) the trial publication rate was minimal, with only 3 trials (9% of all published trials) being published this time point. This is similar to the results of Chapman *et al*. [16] that indicated that the majority of published trials (73%) gets published within 48 months after trial completion. This seems to also agree with Livas *et al*. [23] reporting that only half of study abstracts presented in orthodontic congresses (therefore pertaining to completed studies) reach full publication, with a median presentation-to-publication time of 16 months (1.3 years).

Several registry-, trial-, or patient-related characteristics seemed to affect the publication fate of included registered trials, as seen through the Kaplan-Meier survival estimates and the log-rank tests (Table D in S1 File; Figures B-M in S2 File). For one, considerable differences were observed between trials registered in ClinicalTrials.gov and those registered in ISRCTN, with trials registered in the latter being published less often and later. This might indicate that a true difference exists in the publication of completed trials, but it might also be associated with the considerable geographic differences in the content of the two registries, as ISRCTN included trials primarily from the United Kingdom, while ClinicalTrials.gov had a wider contribution spectrum (Figure N in S2 File).

Counting only unpublished trials with a follow-up period of more than 5 years after trial completion to allow for a free period to be published, indicated that 28% (n = 22) of identified trials still remained unpublished. This is of importance as, based on present results, the chance of those trials being eventually published is small. This represents a waste of resources as well as a gap in the existing research evidence in orthodontics that can have a direct impact on systematic reviews of clinical trials and subsequently on clinical decision making. For example, several trials on new appliances or novel adjunct procedures aimed to expedite orthodontic treatment remain for the present unpublished (Table E in S1 File), which means that existing systematic reviews on this subject [24–27] might not reflect the actual evidence base.

There are several steps that could be implemented toward improvement of trial registration and trial publication practices. Agencies funding clinical trials could implement stricter surveillance systems for trial publication fate and urge researchers to publish the results of their trials, no matter how positive or negative these might seem. Authors are encouraged to register trials prior to trial initiation. In the present study, the majority (76%) of randomized trials were registered retrospectively, something that has also been seen in other fields of medicine [20], but does neither correspond to the notion of *a priori* trial registration nor does it safeguard against selective reporting. Editors of orthodontic journals should avoid judging trial submissions from the attractiveness of their findings and should pursue publication of trials with negative results. Furthermore, editors can indirectly promote trial registration by using it as a prerequisite for a trial to be considered for publication in the journals they manage. The ICMJE changed policy in 2005 to adopt this [7], which resulted in an increase of trial registration [8]. This requirement was later incorporated into the ICMJE’s “uniform requirements for manuscripts submitted to biomedical journals,” along with the updated CONSORT 2010 statement for the reporting of randomized controlled trials [28]. Even though a large part of medical journals requires trials to be registered [29], the landscape in orthodontics still remains bleak: although half (5 out of 10) of orthodontic journals mention registration of randomized clinical trials at their ‘Instructions for authors’ websites, only one (Journal of Orthodontics) words this explicitly as a requirement for publication.

### Strengths and limitations

The strengths of the present study include its comprehensive search of all registered randomized trials in orthodontics and their subsequent publications, as well as the duplicate study selection and data extraction procedures from two independent authors. However, several limitations exist, with first and foremost being the limited number of available registered and completed trials in orthodontics, which affects the study’s statistical power and together with the high number of statistical tests potentially inflates the risk of false findings. Secondly, the present study is dependent upon the reporting quality of the registered trial protocols, which was not always perfect and might even not necessarily reflect the reality. Additionally, although the time from trial completion to publication was based on previously-used date extraction methodology, it is an indirect and possibly imprecise measurement, which does not necessarily agree to a direct follow-up of the trial procedures. Also, the cut-off of 100 patients per trial arm used to categorize trials as large or not large is arbitrary and is not based on a case by case assessment of each outcome, but rather on previous empirical data from general medicine. Finally, we do not know if published trials were previously rejected from any other journals prior to their final publication, which might lead to increased completion-to-publication times.

## Conclusions

The present study within its limitations indicates that around 40% of completed registered randomized trials in orthodontics remains unpublished, while the majority of trials get published in the first 24 to 48 months after completion. However, around one third (28%) of identified completed randomized trials in orthodontics remained unpublished even 5 years after their completion, which could be associated with gaps in the *modus operandi* of orthodontics research and should be taken into account when evaluating the existing orthodontic research.

## Supporting information

S1 File Table A in S1 File. Registry searches performed with identified hits.
Table B in S1 File. List of included and excluded studies with reasons.
Table C in S1 File. Differences in the time from completion to publication for the 33 published trials. (P values correspond to Kruskal-Wallis test).
Table D in S1 File. Influence of protocol or trial publication characteristics on the completion-to-publication survival function of the included registered trial protocols (P values from log-rank test).
Table E in S1 File. Thematology of included and excluded trials’.
(PDF)

S2 File Figure A in S2 File. Kaplan-Meier survival curve for the publication fate of the 80 included randomized trials.
Figure B in S2 File. Kaplan–Meier survival estimates for the lag between trial completion and publication according to registry.
Figure C in S2 File. Kaplan–Meier survival estimates for the lag between trial completion and publication according to registration timing.
Figure D in S2 File. Kaplan–Meier survival estimates for the lag between trial completion and publication according to trial affiliation.
Figure E in S2 File. Kaplan–Meier survival estimates for the lag between trial completion and publication according to geographic origin.
Figure F in S2 File. Kaplan–Meier survival estimates for the lag between trial completion and publication according to sponsor.
Figure G in S2 File. Kaplan–Meier survival estimates for the lag between trial completion and publication according to number of trial centers.
Figure H in S2 File. Kaplan–Meier survival estimates for the lag between trial completion and publication according to trial size.
Figure I in S2 File. Kaplan–Meier survival estimates for the lag between trial completion and publication according to eligible patients’ age.
Figure J in S2 File. Kaplan–Meier survival estimates for the lag between trial completion and publication according to outcome type.
Figure K in S2 File. Kaplan–Meier survival estimates for the lag between trial completion and publication according to journal type.
Figure L in S2 File. Kaplan–Meier survival estimates for the lag between trial completion and publication according to journal type (electronic publishing).
Figure M in S2 File. Kaplan–Meier survival estimates for the lag between trial completion and publication according to their findings.
Figure N in S2 File. Geographic content of the two assessed trial registries.
(PDF)

S1 Dataset Dataset of the current study. (XLSX)
