# Supplementary material for: Randomized clinical trials in orthodontics are rarely registered a priori and often published late or not at all
Source: PLoS One. 2017 Aug 4;12(8):e0182785. doi: 10.1371/journal.pone.0182785 (PMC5544232; doi:10.1371/journal.pone.0182785)
Supplement: S1 File — Table A in S1 File. Registry searches performed with identified hits. Table B in S1 File. List of included and excluded studies with reasons. Table C in S1 File. Differences in the time from completion to publication for the 33 published trials. (P values correspond to Kruskal-Wallis test). Table D in S1 File. Influence of protocol or trial publication characteristics on the completion-to-publication survival function of the included registered trial protocols (P values from log-rank test). Table E in S1 File. Thematology of included and excluded trials’. (PDF) [file pone.0182785.s001.pdf]

Supporting Information file

Randomized clinical trials in orthodontics are rarely registered *a priori* and often published late or not at all

Table A. Registry searches performed with identified hits.

Electronic databases	Search query	Registered trials	Completed trials
ClinicalTrials http://www.clinicaltrials.gov	orthodontic	193	67
ISRCTN https://www.isrctn.com/	orthodontic	73	63

Table B. List of included and excluded studies with reasons.

AA	Source	Trial registration no.	Status
1	CT	NCT01510483	Excluded; non-orthodontic subject
2	CT	NCT02052973	Excluded; non-orthodontic subject
3	CT	NCT02574468	Excluded; non-orthodontic subject
4	CT	NCT01874041	Excluded; non-orthodontic subject
5	CT	NCT00471393	Excluded; non-orthodontic subject
6	CT	NCT02338180	Excluded; non-orthodontic subject
7	CT	NCT01562249	Excluded; non-orthodontic subject
8	CT	NCT02205619	Excluded; non-orthodontic subject
9	IS	ISRCTN00627732	Excluded; non-orthodontic subject
10	IS	ISRCTN02309506	Excluded; non-orthodontic subject
11	IS	ISRCTN10075349	Excluded; non-orthodontic subject
12	IS	ISRCTN10406748	Excluded; non-orthodontic subject
13	IS	ISRCTN10628521	Excluded; non-orthodontic subject
14	IS	ISRCTN11742127	Excluded; non-orthodontic subject
15	IS	ISRCTN13234871	Excluded; non-orthodontic subject
16	IS	ISRCTN14209449	Excluded; non-orthodontic subject
17	IS	ISRCTN16336355	Excluded; non-orthodontic subject
18	IS	ISRCTN16831120	Excluded; non-orthodontic subject
19	IS	ISRCTN20110108	Excluded; non-orthodontic subject
20	IS	ISRCTN20457739	Excluded; non-orthodontic subject
21	IS	ISRCTN23257928	Excluded; non-orthodontic subject
22	IS	ISRCTN31051582	Excluded; non-orthodontic subject
23	IS	ISRCTN42909440	Excluded; non-orthodontic subject
24	IS	ISRCTN52296479	Excluded; non-orthodontic subject
25	IS	ISRCTN55245733	Excluded; non-orthodontic subject
26	IS	ISRCTN55537791	Excluded; non-orthodontic subject
27	IS	ISRCTN64254080	Excluded; non-orthodontic subject
28	IS	ISRCTN65592532	Excluded; non-orthodontic subject
29	IS	ISRCTN72589426	Excluded; non-orthodontic subject
30	IS	ISRCTN76727312	Excluded; non-orthodontic subject
31	IS	ISRCTN84216266	Excluded; non-orthodontic subject
32	IS	ISRCTN57974544	Excluded; non-orthodontic subject
33	CT	NCT00154518	Excluded; no randomized trial
34	CT	NCT02267837	Excluded; no randomized trial
35	CT	NCT02290158	Excluded; no randomized trial
36	CT	NCT01630473	Excluded; no randomized trial
37	CT	NCT00155311	Excluded; no randomized trial
38	CT	NCT00137137	Excluded; no randomized trial
39	CT	NCT02267811	Excluded; no randomized trial
40	CT	NCT03008681	Excluded; no randomized trial
41	CT	NCT01642693	Excluded; no randomized trial
42	CT	NCT00099814	Excluded; no randomized trial
43	CT	NCT00248014	Excluded; no randomized trial
44	CT	NCT02456220	Excluded; no randomized trial
45	CT	NCT02574117	Excluded; no randomized trial
46	CT	NCT02364414	Excluded; no randomized trial
47	IS	ISRCTN66553029	Excluded; no randomized trial
48	IS	ISRCTN47483728	Excluded; no randomized trial
49	IS	ISRCTN70334837	Excluded; problematic registration-publication matching
50	IS	ISRCTN67900267	Included
51	CT	NCT01695928	Included
52	IS	ISRCTN24433142	Included

53	CT	NCT00995436	Included
54	IS	ISRCTN51381850	Included
55	IS	ISRCTN05296896	Included
56	IS	ISRCTN61138858; ISRCTN26364810	Included; two registrations merged
57	IS	ISRCTN79884739	Included
58	CT	NCT02568436	Included
59	IS	ISRCTN38986023	Included
60	CT	NCT01657539	Included
61	CT	NCT01329731	Included
62	IS	ISRCTN66185030	Included
63	IS	ISRCTN27557210	Included
64	CT	NCT00681135	Included
65	IS	ISRCTN52655400	Included
66	CT	NCT01768390	Included
67	CT	NCT01637948	Included
68	CT	NCT01059058	Included
69	CT	NCT02209818	Included
70	CT	NCT02332421	Included
71	CT	NCT02590835	Included
72	CT	NCT01770782	Included
73	CT	NCT02314975	Included
74	CT	NCT02357771	Included
75	CT	NCT01490385	Included
76	CT	NCT00519415	Included
77	IS	ISRCTN05771195	Included
78	IS	ISRCTN10014340	Included
79	IS	ISRCTN76156631	Included
80	CT	NCT02154594	Included
81	CT	NCT02337192	Included
82	CT	NCT02525458	Included
83	IS	ISRCTN04899524	Included
84	CT	NCT02427282	Included
85	CT	NCT01720797	Included
86	IS	ISRCTN32707822	Included
87	CT	NCT02267850	Included
88	CT	NCT01500187	Included
89	CT	NCT01654419	Included
90	CT	NCT03004196	Included
91	IS	ISRCTN14164814	Included
92	IS	ISRCTN35457694	Included
93	CT	NCT00484744	Included
94	CT	NCT02606331	Included
95	CT	NCT02024139	Included
96	CT	NCT01948349	Included
97	CT	NCT01164631	Included
98	CT	NCT02424097	Included
99	CT	NCT02026258	Included
100	CT	NCT02415673	Included
101	CT	NCT00830947	Included
102	IS	ISRCTN29710460	Included
103	CT	NCT01962012	Included
104	IS	ISRCTN05340070	Included
105	CT	NCT02745626	Included

106	IS	ISRCTN33826201	Included
107	IS	ISRCTN61972161	Included
108	IS	ISRCTN80447398	Included
109	CT	NCT01745653	Included
110	CT	NCT01962766	Included
111	IS	ISRCTN68289972	Included
112	CT	NCT02798822	Included
113	CT	NCT01344473	Included
114	IS	ISRCTN38412478	Included
115	IS	ISRCTN46246539	Included
116	IS	ISRCTN53821842	Included
117	CT	NCT02267824	Included
118	CT	NCT02427763	Included
119	CT	NCT01771692	Included
120	IS	ISRCTN97142521	Included
121	IS	ISRCTN79526387	Included
122	CT	NCT00213434	Included
123	IS	ISRCTN22535947	Included
124	IS	ISRCTN41545651	Included
125	IS	ISRCTN75477546	Included
126	IS	ISRCTN83971890	Included
127	IS	ISRCTN14198853	Included
128	IS	ISRCTN56295329	Included
129	IS	ISRCTN56613406	Included

CT, ClinicalTrials.gov register; IS, ISRCTN register.

Table C. Differences in the time from completion to publication for the 33 published trials. (P values correspond to Kruskal-Wallis test)

Factor	Category	N[a]	Median (IQR)	P value
Registry	ClinicalTrials.gov	20	12.9 (7.7-22.7)	0.001
	ISRCTN	13	31.6 (27.1-46.7)	
Sponsor	Internal	14	10.6 (8.3-17.3)	0.05
	Government	10	30.5 (23.8-42.3)	
	Commercial	3	29.3 (13.8-36.6)	
	Other	6	26.4 (7.0-29.0)	
Affiliation	University	26	16.5 (9.0-29.4)	0.15
	Private practice	0		
	Hospital	7	29.3 (13.3-62.0)	
Geographical origin	Europe	20	28.4 (14.2-39.5)	0.11
	Asia	8	10.5 (5.4-15.5)	
	North America	2	19.7 (14.2-25.2)	
	South America & Africa		20.1 (8.3-29.0)	
Registration	Prospective	7	13.8 (9.0-31.6)	0.57
	Retrospective	26	24.5 (9.1-32.5)	
Outcome	Objective	31	20.1 (9.1-31.6)	0.65
	Subjective	2	37.0 (9.0-65.0)	
Multicenter	Yes	2	38.0 (29.3-46.7)	0.17
	No	31	17.3 (9.0-31.6)	
Patients	Children	14	30.5 (15.6-55.8)	0.009
	Adults	2	7.6 (1.8-13.3)	
	Mixed	15	13.8 (7.0-25.2)	
	Not specified	2	29.8 (27.1-32.5)	
Large (≥ 100 patients)	Yes	4	47.8 (23.3-64.5)	0.05
	No	28	16.5 (9.0-29.4)	
Journal	Specialty	21	27.5 (15.1-42.3)	0.005
	Non-specialty	12	10.5 (5.7-18.7)	
Journal with Epub	Yes	18	17.9 (8.3-32.5)	0.49
	No	15	23.8 (13.3-31.6)	
Findings	Positive	15	14.2 (7.0-29.0)	0.11
	Non positive	14	26.2 (9.1-36.6)	
	Unclear	4	37.4 (22.3-53.2)	

IQR, interquartile range; ISRCTN, International Standard Randomised Controlled Trial Number.

Table D. Influence of protocol or trial publication characteristics on the completion-to-publication survival function of the included registered trial protocols (P values from log-rank test).

Factor	N[a]	Significant	P value
Register (ClinicalTrials.gov or ISRCTN)	80	*	0.008*
Sponsor (internal, government, external commercial, other)	80		0.37
Trial affiliation (university, practice or hospital)	80		0.71
Geographic (Europe, Asia, N. America, or S. America/Africa)	80	*	<0.001**
Registration timing (prospective or retrospective)	80		0.62
Outcome (patient-reported or doctor-assessed)	79		0.48
Multicenter (yes or no)	80		0.48
Patients (children, adults, mixed or unspecified)	80	*	0.008
Journal type (specialty or non-specialty)	33	*	0.002
Journal epub (yes or no)	33		0.55
Trial size (large or not large)	71		0.81
Trial results (positive or not-positive/unclear)	33		0.30

ISRCTN, International Standard Randomised Controlled Trial Number.

Table E. Thematology of included and excluded trials'.

No	Trial ID	Thematology	Title
		Published trials	
1	ISRCTN76156631	Adhesives	Comparing bond failure in direct and indirect bonding techniques
2	NCT02568436	Adjunct; laser	Effect of Low Level Laser Therapy in Accelerating Tooth Movement For Dental Crowding Cases
3	NCT02209818	Adjunct; laser	Effect of Laser Application in Pain Management Following the Use of Orthodontic Elastomeric Separators
4	NCT01490385	Adjunct; phototherapy	The Effect of Light Emitting Diode Phototherapy on the Rate of Orthodontic Tooth Movement - A Clinical Study
5	NCT02337192	Adjunct; phototherapy	Antimicrobial Photodynamic Therapy Applied in Orthodontic. Orthodontic Patients.
6	NCT01695928	Adjunct; shockwave	Effect of Shock Waves on Tooth Movement and Miniscrew Stability
7	NCT02590835	Adjunct; surgical orthodontics	Efficiency of Piezocision-assisted Orthodontic Treatment in Adult Patients
8	NCT02314975	Adjunct; vibration	Intermittent Vibrational Force and Orthodontic Tooth Movement
9	ISRCTN27557210	Bactereamia	An investigation of odontogenic bacteraemia associated with orthodontic treatment procedures
10	NCT02332421	Canine retraction	The Use of a Modified Dentoalveolar Distractor to Retract Maxillary Canines
11	ISRCTN61138858; ISRCTN26364810	Class II	The long-term effects of orthodontic growth modification for severe Class II anomalies
12	ISRCTN52655400	Class II	Prospective comparative study of affects of two functional appliances
13	NCT00519415	Class III	Comparative Results of Maxillary Deficiency Treatment by Tongue Plate and Facemask in Growing Patients
14	ISRCTN10014340	Class III	Early orthodontic treatment for reverse bite: an orthopaedic facemask treatment for children < 10 years old reduces the need for jaw surgery in the late teens
15	ISRCTN79884739	Chewing gum	Chewing gum and orthodontic pain
16	NCT01637948	Mouthwash; complementary medicine	Controlled Clinical Trial of Traditional Chinese Medicine Mouthrinse
17	ISRCTN38986023	Orthognathic	A prospective randomised clinical trial to assess change in nasal soft tissue dimensions in orthognathic surgery evaluated using a 3D imaging system
18	NCT01770782	Retention; RME	Orthodontic Retention on the Maxillary Stability After SARME Using Laser Scanner
19	ISRCTN67900267	Brackets; self-ligating	A prospective randomized trial investigating lower incisor inclination and mandibular arch dimensional changes of two pre-adjusted edgewise orthodontic bracket systems in non-extraction cases
20	ISRCTN51381850	Brackets; self-ligating	A randomised controlled trial comparing conventional, active and passive self-ligating orthodontic bracket systems
21	ISRCTN05296896	Brackets; self-ligating	A prospective randomised clinical trial to compare pain levels of two orthodontic fixed bracket systems
22	ISRCTN66185030	Brackets; self-ligating	A prospective randomized trial investigating the alignment efficiency of two pre-adjusted edgewise orthodontic bracket systems
23	ISRCTN05771195	Space closure	An investigation of two methods of orthodontic space closure: nickel titanium versus stainless steel springs
24	ISRCTN24433142	TADs; reinforcement	Palatal implants versus headgear for orthodontic anchorage - a randomised controlled trial
25	NCT00995436	TADs; reinforcement	Chesterfield Micro-implant Study Involving Three Types of Anchorage Methods in Orthodontics
26	NCT01657539	Caries prevention	Effect of Probiotics Containing Yogurts on the Composition of Biofilms in Patients Under Orthodontic Treatment
27	NCT00681135	Caries prevention	Mechanical Plaque Control and Gingivitis Reduction in Fixed Appliance Patients
28	NCT01768390	Caries prevention	Caries-preventive Effect of a Dentifrice Containing 5,000 Ppm Fluoride in Orthodontic Patients
29	NCT02357771	Caries prevention	Probiotic Lozenge Reduce Streptococcus Mutans in Plaque in Orthodontic Bracket Patients
30	NCT02154594	Caries prevention	"The Efficacy of Acacia Catechu Mouthrinse as Antiplaque and Antigingivitis Agent in Fixed Orthodontic Appliance Patients.
31	NCT02525458	Caries prevention	Quaternary Ammonium Methacryloxy Silicate-containing Acrylic Resin
32	NCT01329731	Caries treatment	Remineralisation of White Spot Lesions by Elmex® gelée in Post-orthodontic Patients

33	NCT01059058	Caries treatment	Study of Treatments Used for White Spot Lesions After Orthodontic Treatment.
			Unpublished trials
1	ISRCTN35457694	Adhesives	A randomised controlled trial investigating the orthodontic bracket bond failure rates using Ortho Solo Universal bond enhancer compared to a conventional bonding primer
2	ISRCTN05340070	Adhesives	The effect of a fluoride releasing, non-primer step required, no mix composite: A clinical trial
3	ISRCTN61972161	Adhesives	A randomised prospective clinical trial to compare two different methods of applying a new resin-reinforced chemically-cured "moisture-friendly" glass ionomer cement for the bonding of orthodontic brackets
4	ISRCTN41545651	Adhesives	A randomised clinical trial to compare bond failure rates with and without the use of Ortho Solo
5	NCT01745653	Adjunct; laser	Effect of Low Level Laser Therapy on Pain Occurring After Insertion of an Orthodontic Device
6	NCT02267850	Adjunct; phototherapy	Effect of Intra-Oral Photobiomodulation on Orthodontic Treatment Time
7	NCT02267824	Adjunct; phototherapy	The Effects of Extraoral Photobiomodulation on the Rate of Orthodontic Alignment
8	NCT01720797	Adjunct; surgical orthodontics	Alveolar Microperforation for Inflammation-Enhanced Tooth Movement During Orthodontic Treatment
9	NCT02606331	Adjunct; surgical orthodontics	Efficacy of Minimally Invasive Surgical Technique in Accelerating Orthodontic Treatment
10	NCT02026258	Adjunct; surgical orthodontics	Efficiency of Piezotome-Corticision Assisted Orthodontics
11	NCT00830947	Adjunct; vibration	Effect of Cyclic Loading (Vibration) on Orthodontic Tooth Movement
12	NCT01962012	Adjunct; vibration	Effect of AcceleDent® Aura on Orthodontic Tooth Movement With Aligners
13	ISRCTN14164814	Aligners	Analysis of gingival fluids of orthodontic patients undergoing different treatments
14	ISRCTN33826201	Aligners	Invisalign - a concept for the prevention of white spot lesions in teenaged orthodontic patients?
15	NCT02427763	Aligners	Microbiological and Epithelial Evaluation Related to the Use of Orthodontic Thermoplastic Device
16	ISRCTN32707822	Bands	Application of molecular diagnostic methodologies to analyse plaque microbial population shifts associated with placement of fixed orthodontic appliances
17	NCT01654419	Class II	Comparison of the Quantity of Distal Movement of the Upper Canine and Molar
18	ISRCTN80447398	Class II	Comparison between twin-block & forsus functional appliances
19	NCT02024139	Chewing gum	Efficacy of Chewing Sugarless Gum for Reduction of Orthodontic Appliance Pain
20	ISRCTN46246539	Impacted teeth	Exposure of palatal canines: cover-plate vs periodontal dressing
21	ISRCTN83971890	Impacted teeth	An investigation into the management of interrupted incisor teeth
22	NCT01771692	Ligatures	A Randomised Clinical Trial: Conventional Ligation vs Figure of 8
23	NCT00484744	Pain medication	Acetaminophen Versus Ibuprofen for the Control of Immediate and Delayed Pain Following Orthodontic Separator Placement
24	NCT01962766	Myofunctionnal therapy	Myofunctionnal Therapy on Nasal Breathing and Orthodontic Corrections Stability
25	NCT00213434	OSAS	Evaluation of a Mandibular Advancement Device in the Treatment of Obstructive Sleep Apnea Syndrome
26	ISRCTN14198853	Patient information	Do information leaflets entitled "your first visit to the orthodontist" help to improve knowledge of, and satisfaction with, the initial orthodontic consultation
27	ISRCTN22535947	Retention	The benefit of prolonged orthodontic retention - a randomised clinical trial
28	ISRCTN56295329	Retention	How should patients be supervised during orthodontic retention? A randomised controlled trial
29	NCT02798822	RME	Maxillary and Mandibular Arch Response to RME: a Multicentric Randomized Controlled Trial
30	ISRCTN53821842	RME; CLP	Is preliminary orthodontic treatment of benefit to the success of alveolar bone grafting?
31	NCT01948349	Brackets; self-ligating	Impact of Bracket Design and Oral Hygiene Maintenance on Halitosis in the Orthodontic Patient
32	ISRCTN68289972	Brackets; self-ligating	A comparison of mandibular archwidth changes using two different bracket systems
33	ISRCTN56613406	Brackets; self-ligating	Self-ligating brackets and elastomeric rings - a comparison of orthodontic ligation techniques on patient oral hygiene and microbial colonisation

34	NCT02745626	Brackets; self-ligating / aligners	Comparison of Oral Hygiene & Root Resorption During Orthodontic Treatment
35	ISRCTN79526387	Smoking	A randomised controlled trial to assess whether smoking cessation instruction provided by orthodontists to teenage orthodontic patients is beneficial
36	NCT02427282	TADs; distalization	Distalization by Miniscrew
37	ISRCTN29710460	TADs; reinforcement	Are microimplants as effective at providing orthodontic anchorage as conventional methods
38	ISRCTN97142521	TADs; reinforcement	Early Loading of Palatal Implants (ortho-type II) a prospective multicenter randomised controlled clinical trial
39	NCT01164631	Tonsilitis	Pharyngeal Size in Patients With Obstructive Tonsils Under Orthodontic Treatment
40	NCT02415673	Wires	Alignment Efficiency of Two Thermal Activated Orthodontic Archwire Sequences
41	ISRCTN38412478	Wires	A comparison of the tooth movements achieved with two aligning archwires
42	ISRCTN75477546	Wires	A randomised controlled clinical trial comparing aesthetic arch wires and nickel titanium arch wires in initial alignment
43	ISRCTN04899524	Caries prevention	An in situ study to determine the effects of calcium-based toothpaste in orthodontic patients
44	NCT03004196	Caries prevention	Comparison of Efficacy Of Probiotic Toothpaste and Chlorhexidine Mouthwash To Reduce S.Mutans
45	NCT01500187	Caries treatment	Fluoride Varnish for Treatment of White Spot Lesions
46	NCT01344473	Caries treatment	A Trial of Tooth Mousse to Remineralise Post-orthodontic Treatment White Spot Lesions
47	NCT02424097	Caries treatment/prevention	MI Varnish and MI Paste Plus in a Caries Prevention and Remineralization Study

.
