# Supplementary material for: Randomized clinical trials in orthodontics are rarely registered a priori and often published late or not at all
Source: PLoS One. 2017 Aug 4;12(8):e0182785. doi: 10.1371/journal.pone.0182785 (PMC5544232; doi:10.1371/journal.pone.0182785)

Fig A. Kaplan-Meier survival curve for the publication fate of the 80 included randomized trials.

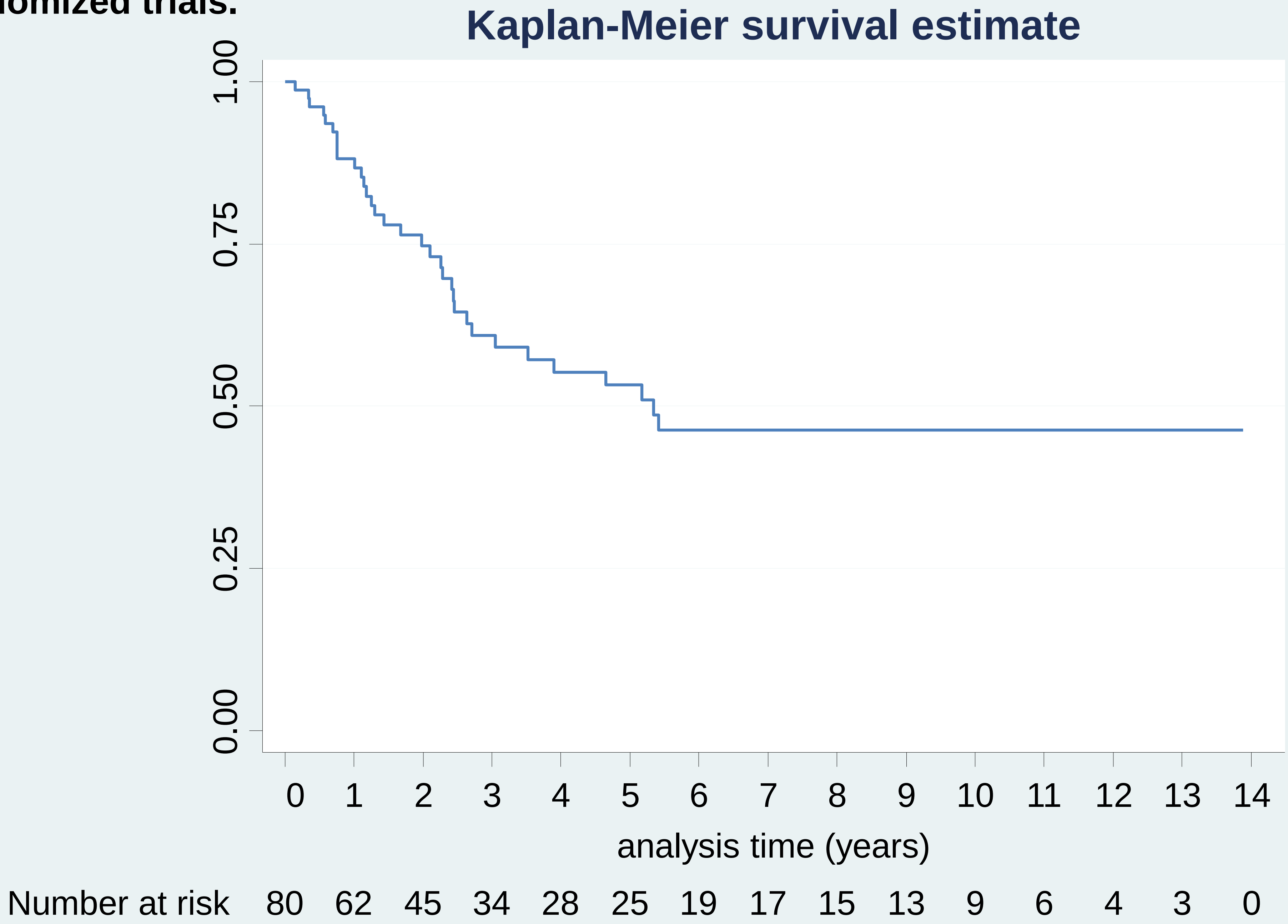

Fig B. Kaplan–Meier survival estimates for the lag between trial completion and publication according to registry.

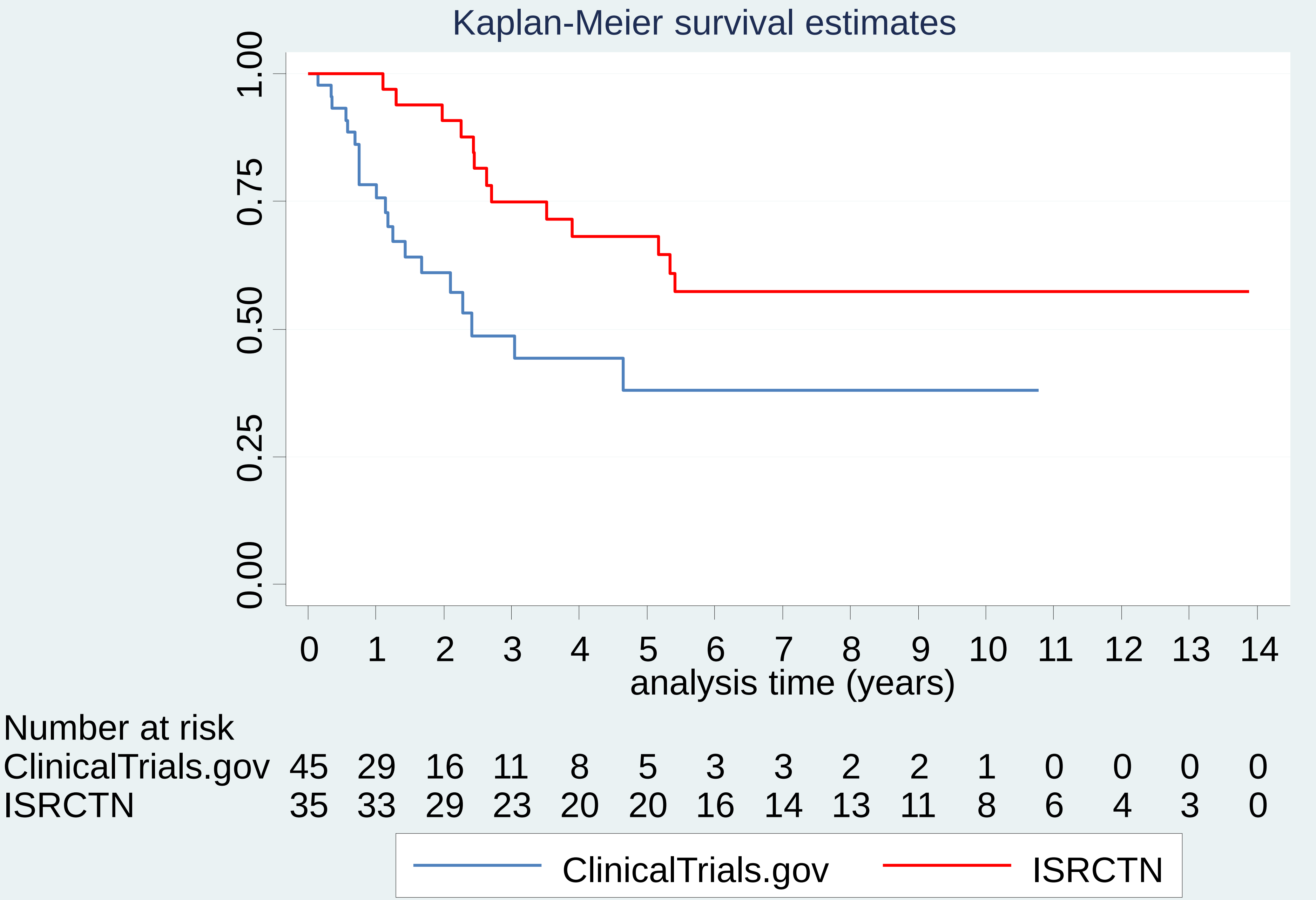

Fig C. Kaplan–Meier survival estimates for the lag between trial completion and publication according to registration timing.

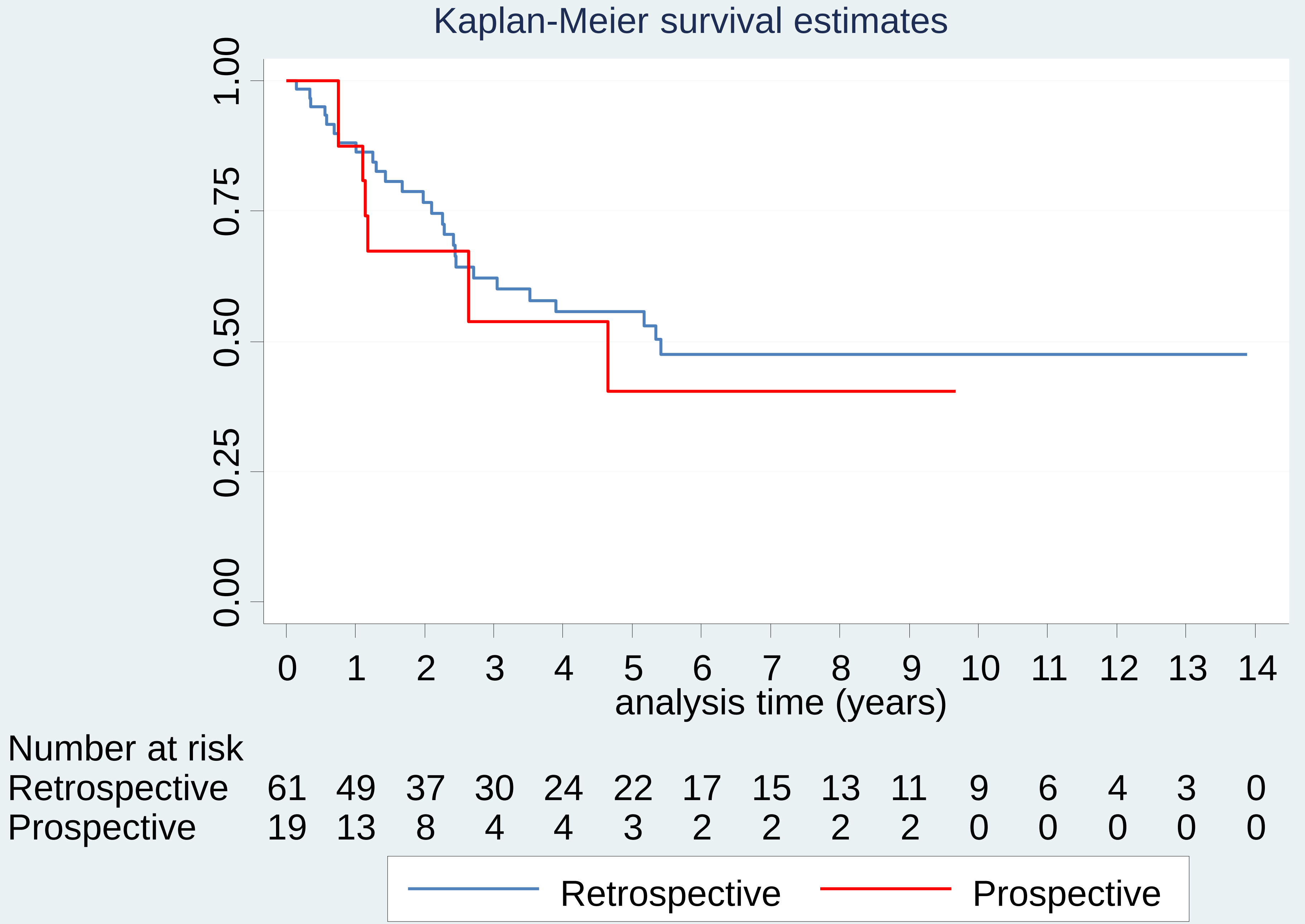

Fig D. Kaplan–Meier survival estimates for the lag between trial completion and publication according to trial affiliation.

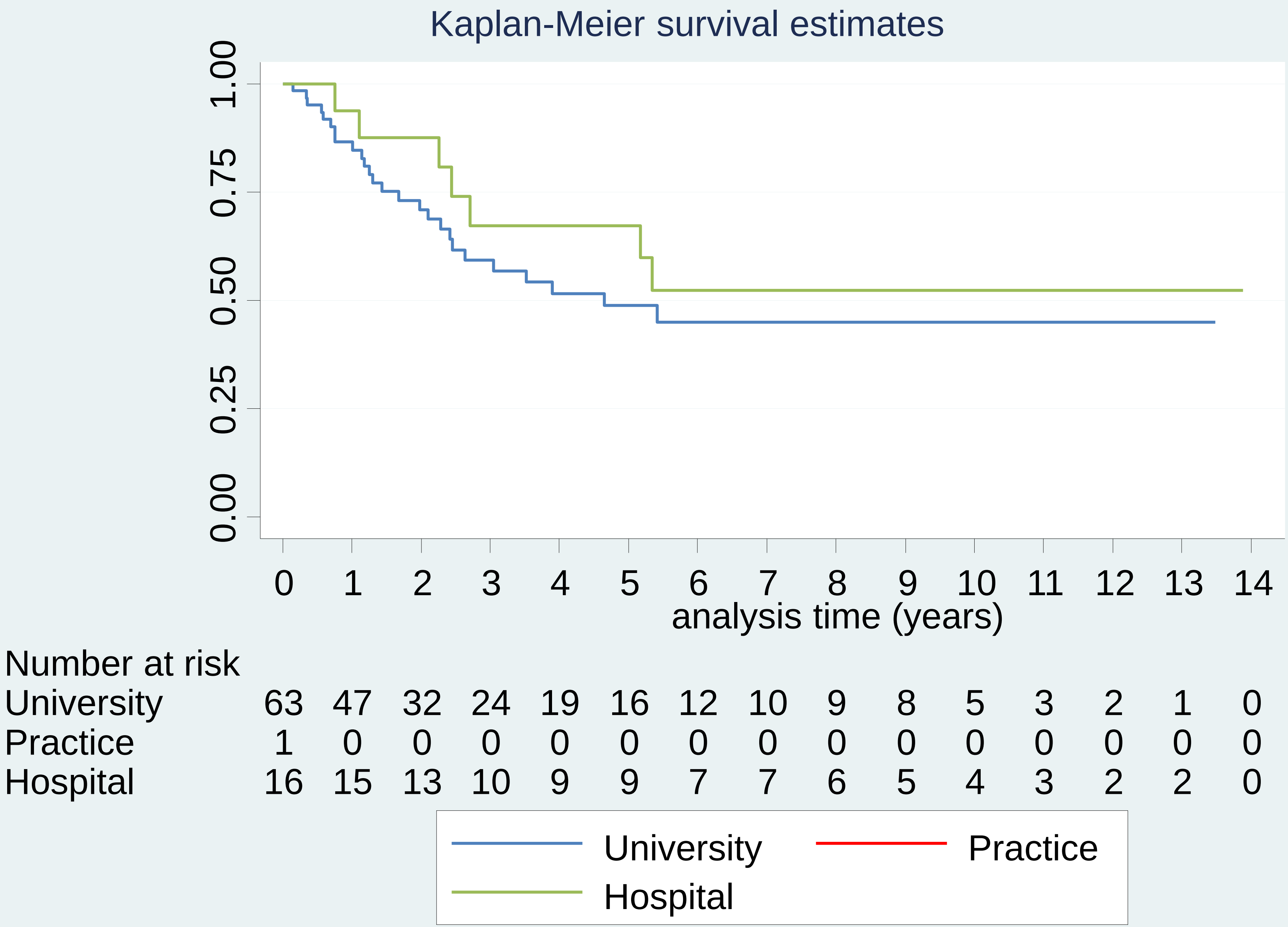

Fig E. Kaplan–Meier survival estimates for the lag between trial completion and publication according to geographic origin.

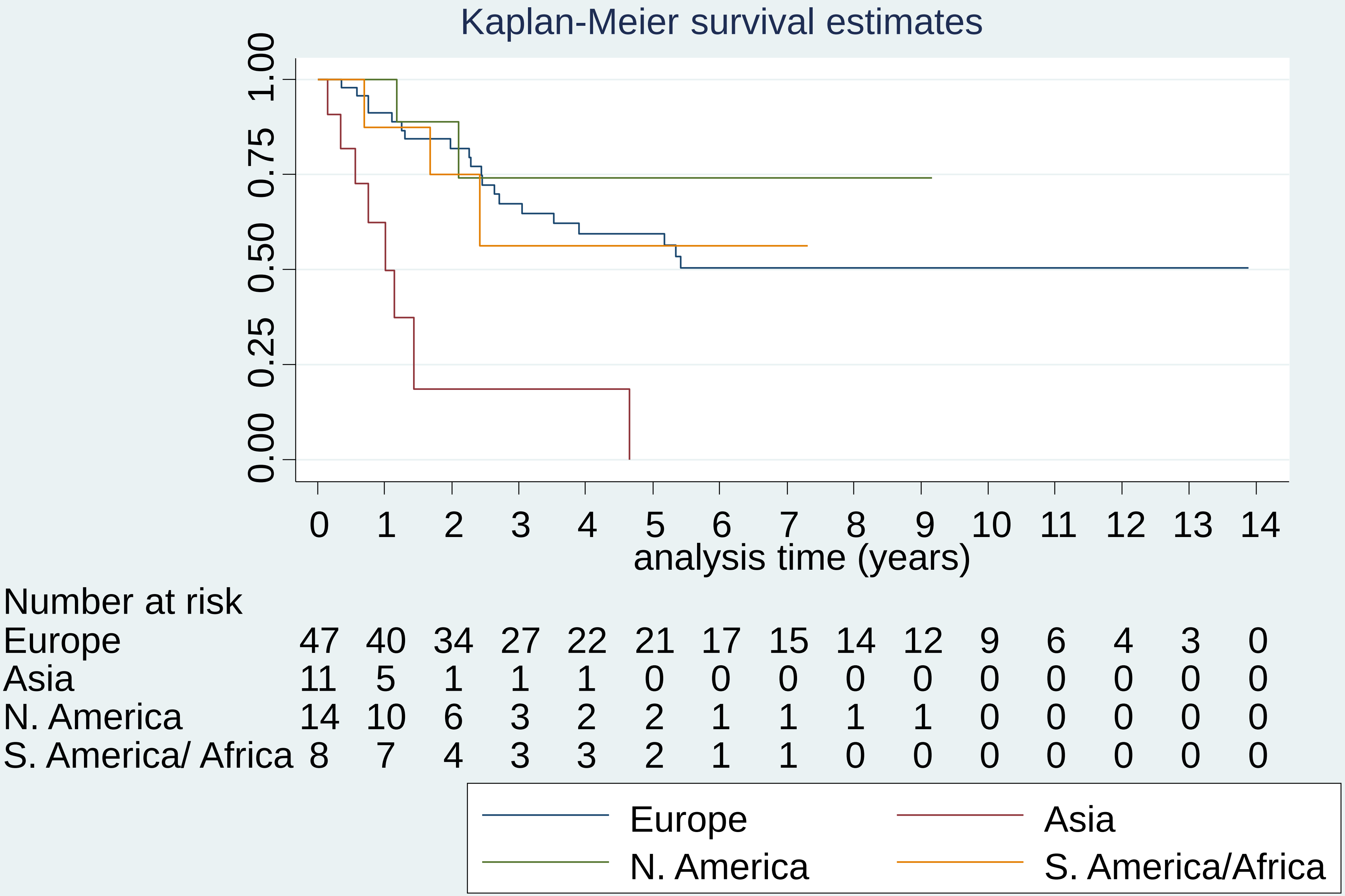

Fig F. Kaplan–Meier survival estimates for the lag between trial completion and publication according to sponsor.

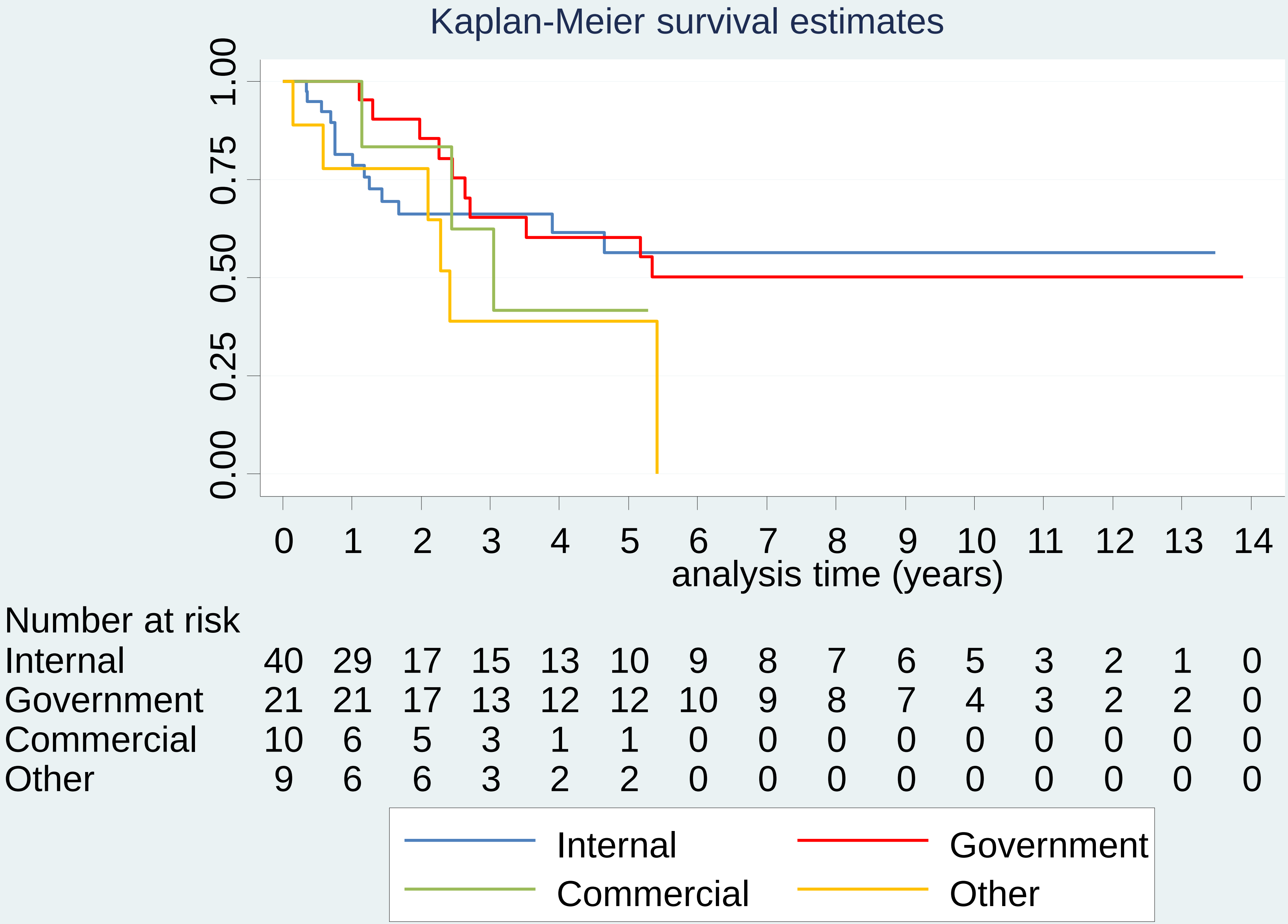

Fig G. Kaplan–Meier survival estimates for the lag between trial completion and publication according to number of trial centers.

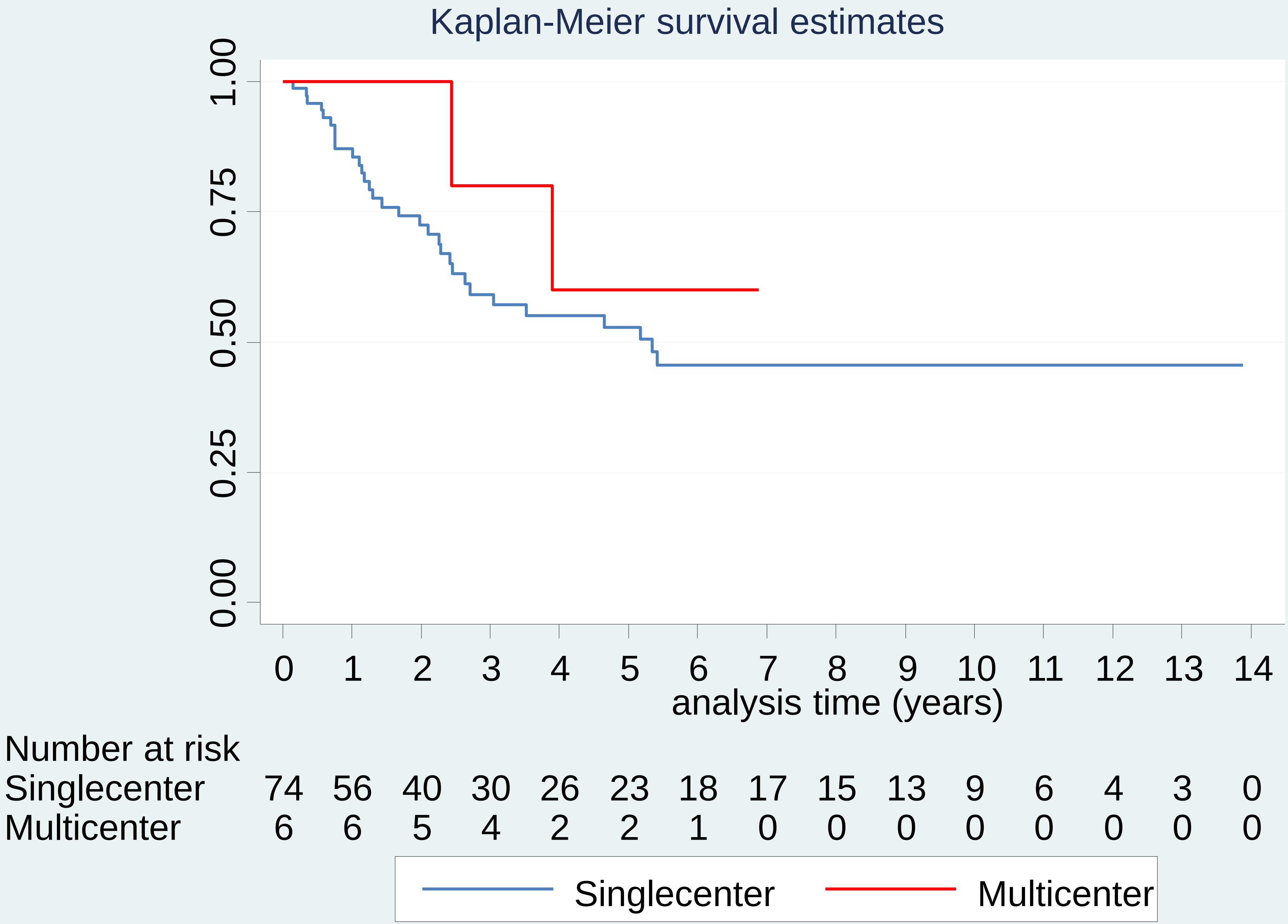

Fig H. Kaplan–Meier survival estimates for the lag between trial completion and publication according to trial size.

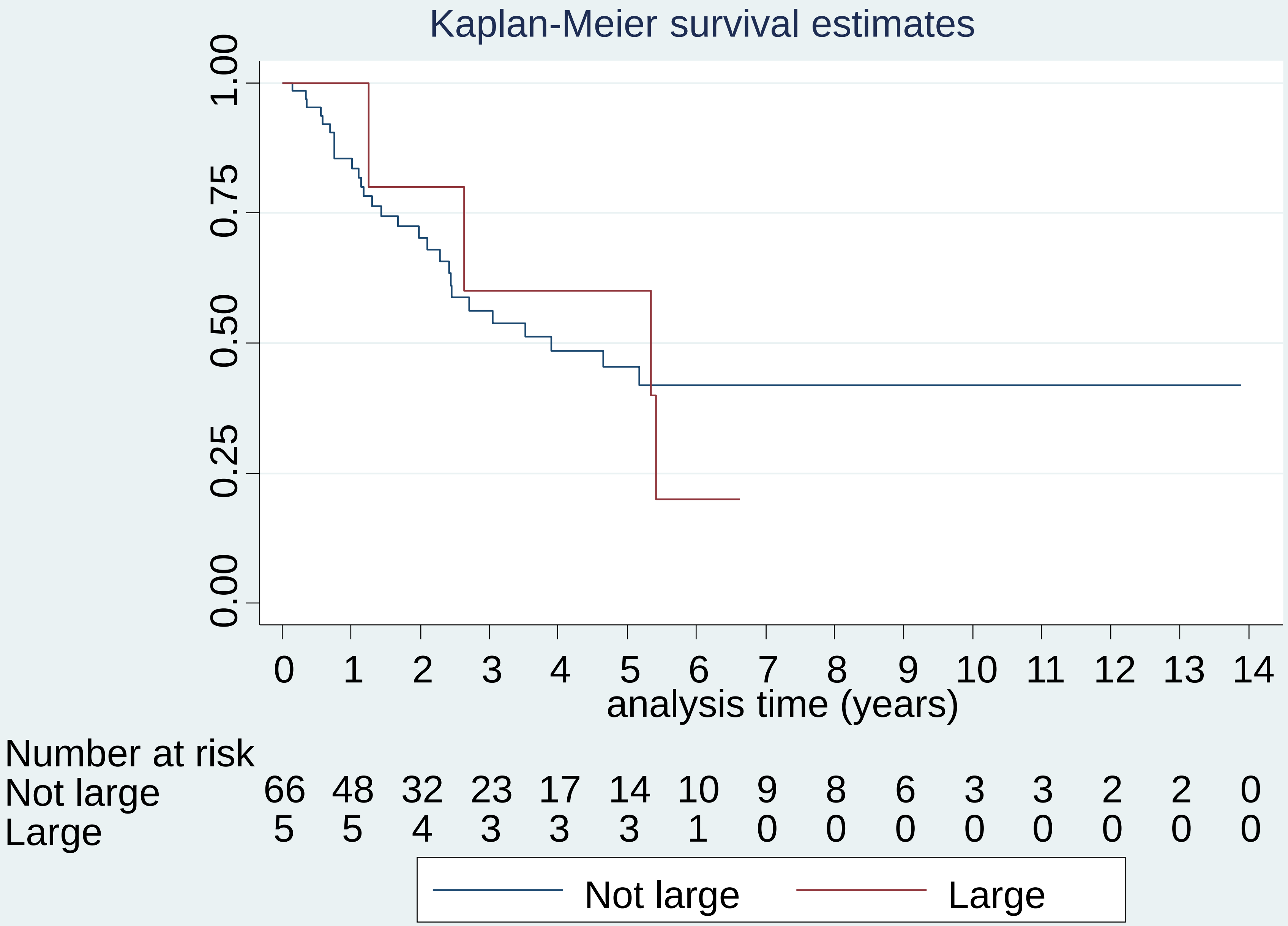

Fig I. Kaplan–Meier survival estimates for the lag between trial completion and publication according to eligible patients' age.

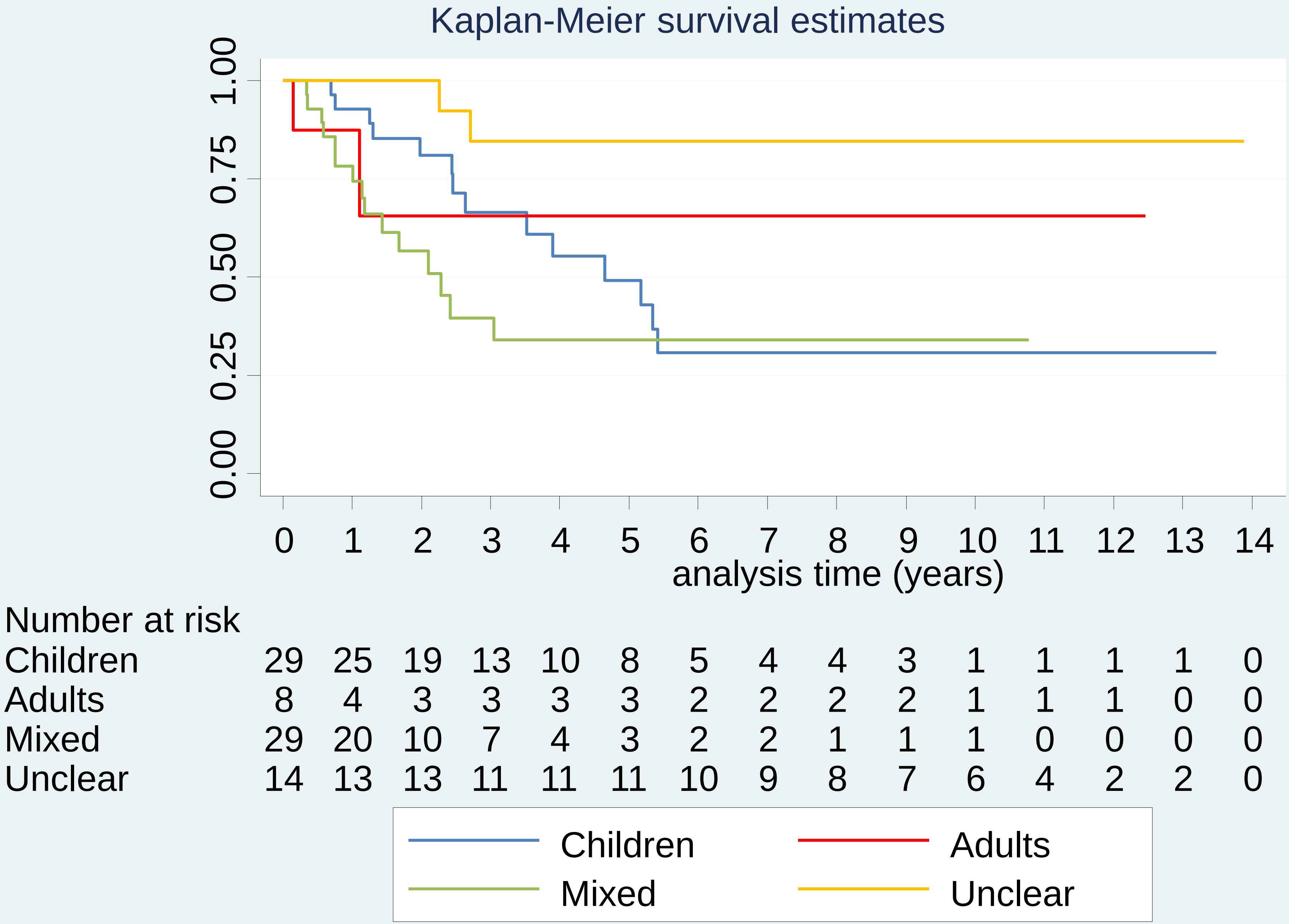

Fig J. Kaplan–Meier survival estimates for the lag between trial completion and publication according to outcome type.

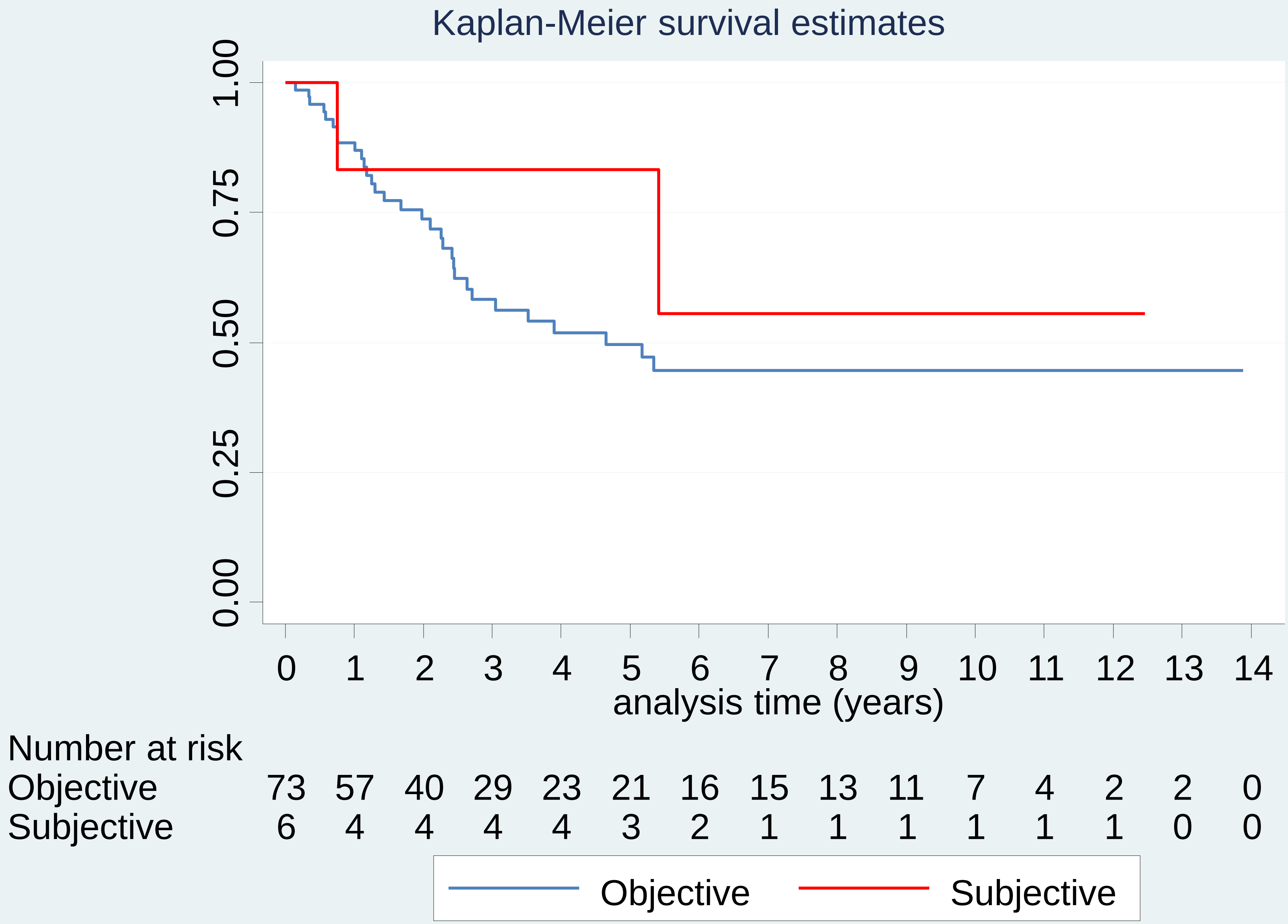

Fig K. Kaplan–Meier survival estimates for the lag between trial completion and publication according to journal type.

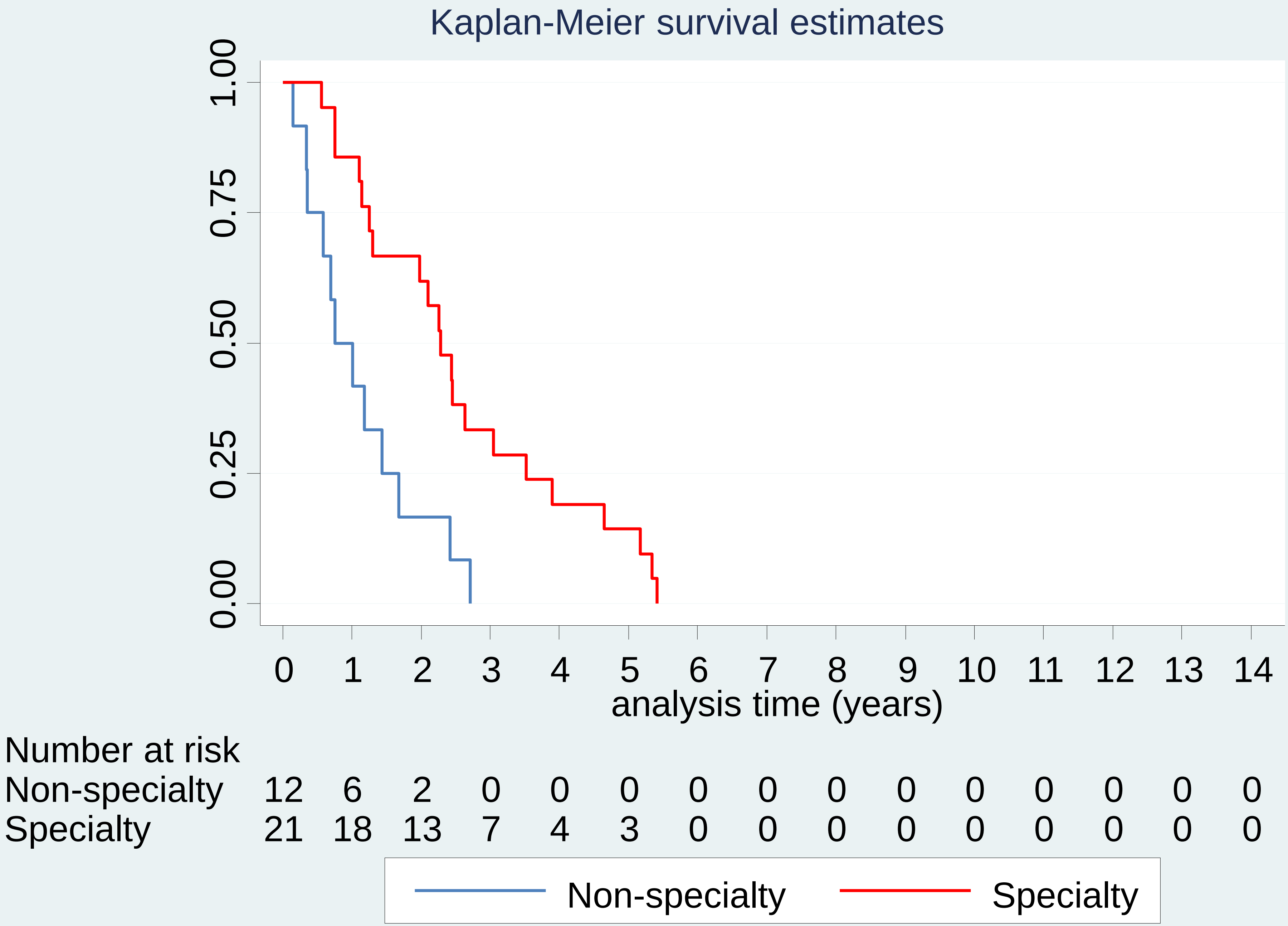

Fig L. Kaplan–Meier survival estimates for the lag between trial completion and publication according to journal type (electronic publishing).

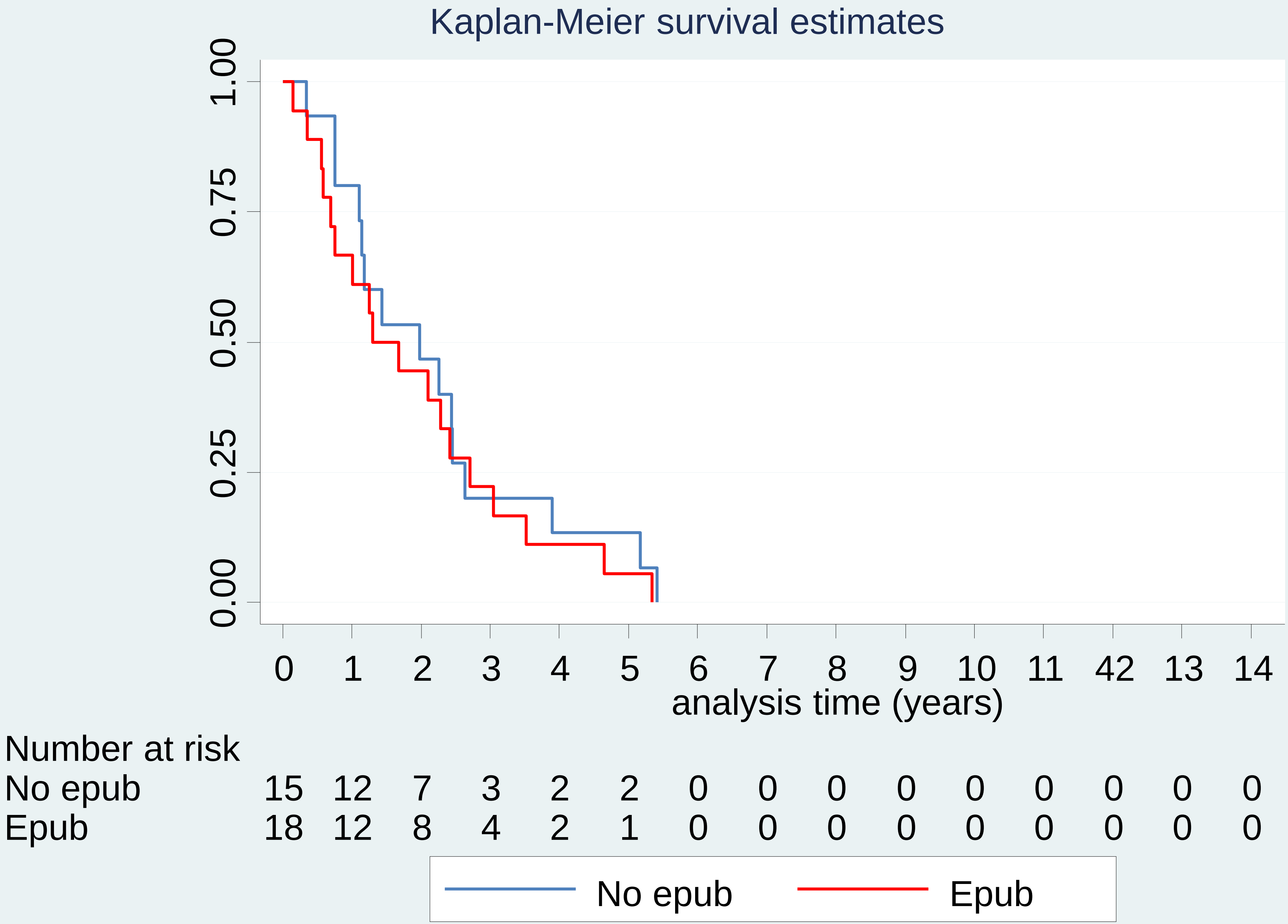

Fig M. Kaplan–Meier survival estimates for the lag between trial completion and publication according to their findings.

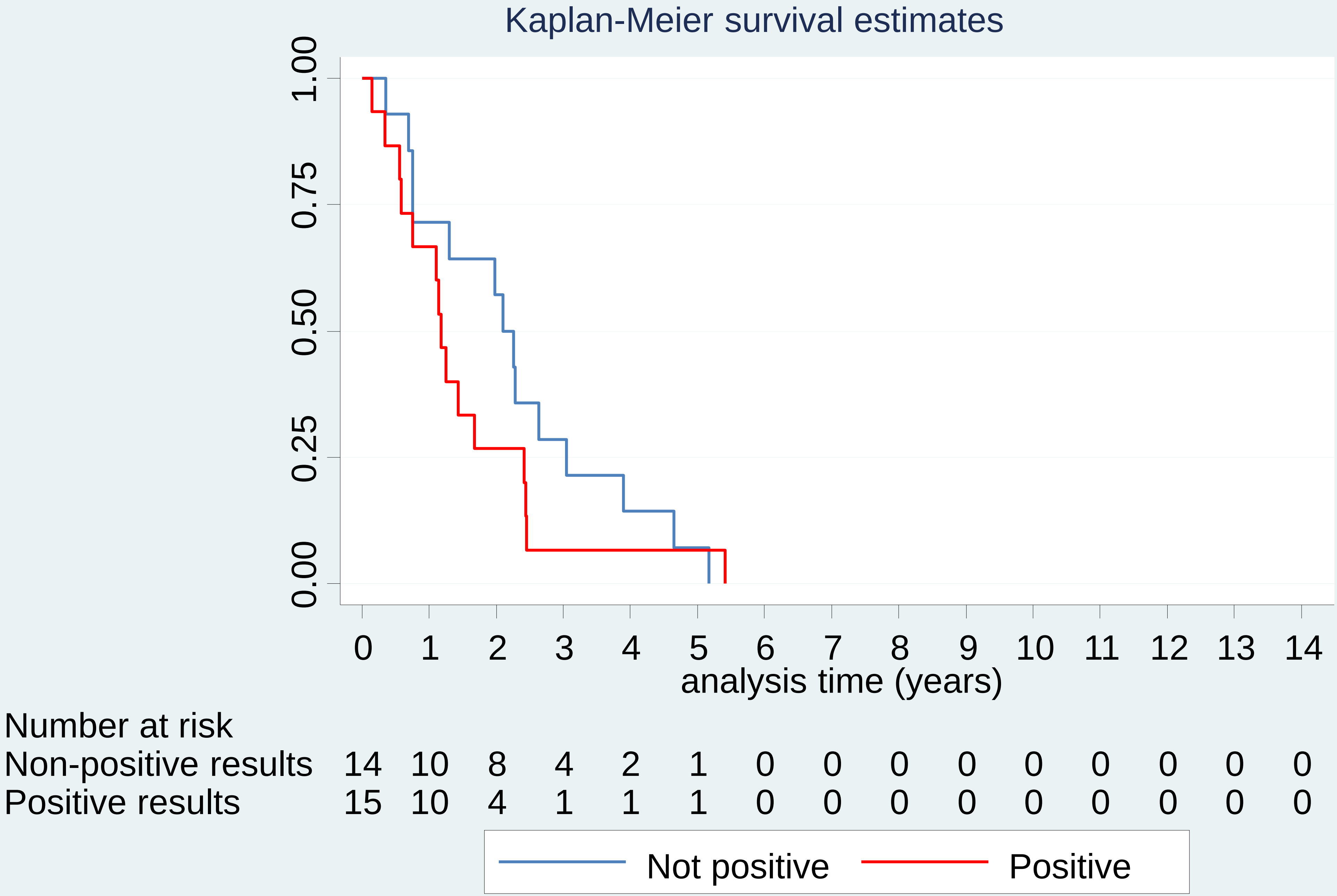

Fig N. Geographic content of the two assessed trial registries.

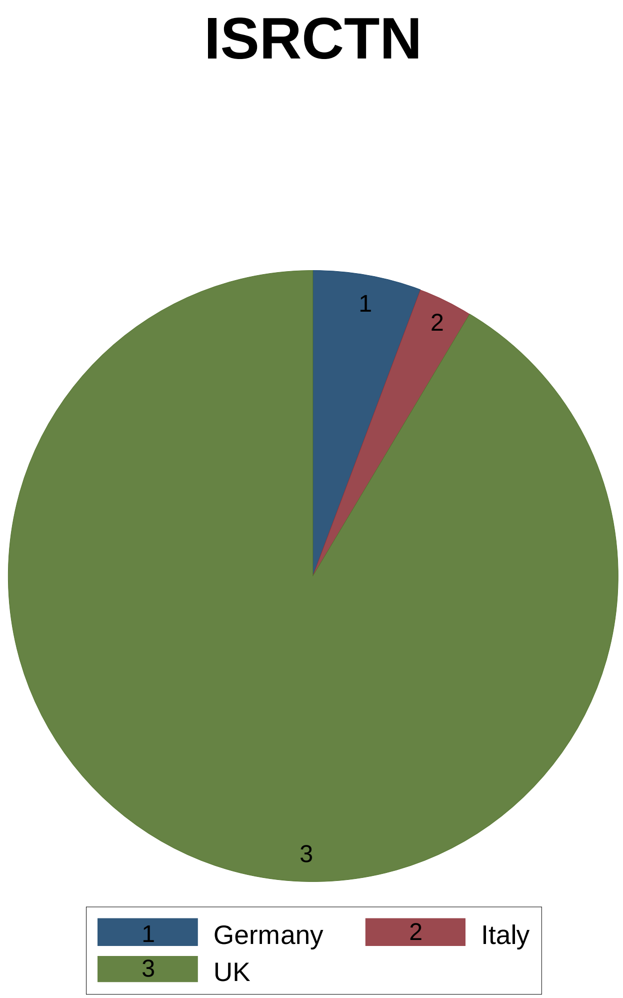

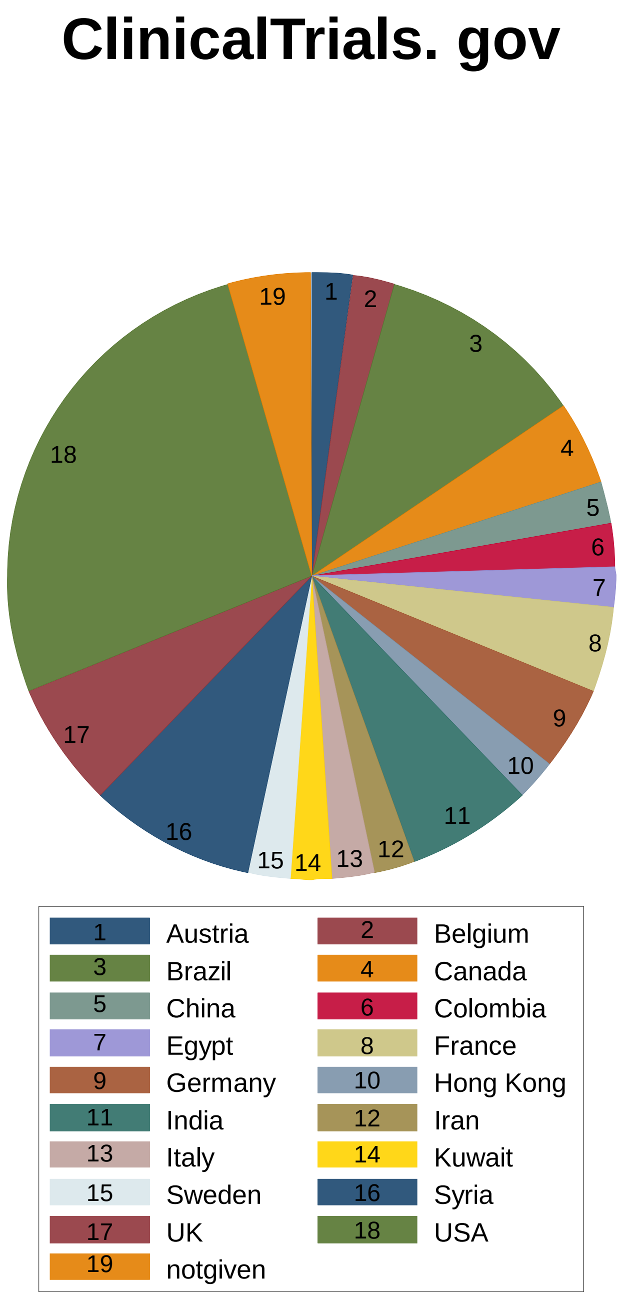

Supplement: S2 File — Figure A in S2 File. Kaplan-Meier survival curve for the publication fate of the 80 included randomized trials. Figure B in S2 File. Kaplan–Meier survival estimates for the lag between trial completion and publication according to registry. Figure C in S2 File. Kaplan–Meier survival estimates for the lag between trial completion and publication according to registration timing. Figure D in S2 File. Kaplan–Meier survival estimates for the lag between trial completion and publication according to trial affiliation. Figure E in S2 File. Kaplan–Meier survival estimates for the lag between trial completion and publication according to geographic origin. Figure F in S2 File. Kaplan–Meier survival estimates for the lag between trial completion and publication according to sponsor. Figure G in S2 File. Kaplan–Meier survival estimates for the lag between trial completion and publication according to number of trial centers. Figure H in S2 File. Kaplan–Meier survival estimates for the lag between trial completion and publication according to trial size. Figure I in S2 File. Kaplan–Meier survival estimates for the lag between trial completion and publication according to eligible patients’ age. Figure J in S2 File. Kaplan–Meier survival estimates for the lag between trial completion and publication according to outcome type. Figure K in S2 File. Kaplan–Meier survival estimates for the lag between trial completion and publication according to journal type. Figure L in S2 File. Kaplan–Meier survival estimates for the lag between trial completion and publication according to journal type (electronic publishing). Figure M in S2 File. Kaplan–Meier survival estimates for the lag between trial completion and publication according to their findings. Figure N in S2 File. Geographic content of the two assessed trial registries. (PDF) [file pone.0182785.s002.pdf]
